# Saline immersion snare-tip coagulation for post procedural bleeding prevention after endoscopic papillectomy

**DOI:** 10.1055/a-2825-8695

**Published:** 2026-03-16

**Authors:** Marco Spadaccini, Vincenzo Vadala', Matteo Colombo, Davide Massimi, Antonio Capogreco, Cesare Hassan, Alessandro Repici

**Affiliations:** 1 9268 Gastroenterology and Digestive Endoscopy Unit, IRCCS Humanitas Research Hospital, Rozzano, Milan, Italy; 2 437807 Department of Biomedical Sciences, Humanitas University, Pieve Emanuele, Milan, Italy

Post-procedural bleeding (PPB) is the most frequent complication after endoscopic papillectomy 1 , with rates substantially higher than those reported after endoscopic resection of pre-neoplastic lesions in other gastrointestinal sites. Although prophylactic closure of the mucosal defect with clips has proven effective in reducing PPB after upper and lower gastrointestinal endoscopic resections 2 3 4 , complete closure of the papillectomy bed is usually not feasible because of the presence of the biliary and pancreatic orifices. For this reason, alternative strategies for bleeding prevention are needed.

In the colorectum, prophylactic vessel coagulation performed in a saline-immersion environment using high-voltage coagulation current delivered through the snare tip has been shown to reduce delayed bleeding 5 . This technique maximizes the coagulation effect while minimizing the risk of unintended cutting of exposed vessels.

We report the case of a 79-year-old woman who underwent endoscopic papillectomy for an ampullary adenoma with high-grade dysplasia. At the end of the resection, prophylactic saline-immersion coagulation was performed to prevent delayed bleeding ( Video 1 ). The snare tip was gently placed in contact with visible vessels, and high-voltage coagulation current (Forced Coag, Effect 3.0, Erbe VIO 3) was applied, resulting in progressive presealing. A single unintended spark occurred due to small air bubbles, producing a transient cutting effect and highlighting the importance of continuous flushing during the procedure. No delayed bleeding or other adverse events were observed during 30 days of follow-up.

Saline immersion snare-tip coagulation with high-voltage current after endoscopic papillectomy allowed successful pre-sealing of large submucosal vessels at the resection bed.Video 1

This case illustrates the potential role of saline-immersion snare-tip coagulation as a simple and effective adjunctive method for PPB prevention after endoscopic papillectomy.

Endoscopy_UCTN_Code_TTT_1AO_2AD Endoscopy_UCTN_Code_TTT_1AO_2AN
